# Risk factors for early metachronous tumor development after endoscopic resection for early gastric cancer

**DOI:** 10.1371/journal.pone.0185501

**Published:** 2017-09-26

**Authors:** Jae Yong Park, Sang Gyun Kim, Jung Kim, Seung Jun Han, Sooyeon Oh, Ji Min Choi, Joo Hyun Lim, Hyunsoo Chung, Hyun Chae Jung

**Affiliations:** 1 Department of Internal Medicine, Chung-Ang University Hospital, Seoul, Korea; 2 Department of Internal Medicine and Liver Research Institute, Seoul National University College of Medicine, Seoul, Korea; 3 Department of Internal Medicine, Healthcare Research Institute, Seoul National University Hospital Healthcare System Gangnam Center, Seoul, Korea; University Hospital Llandough, UNITED KINGDOM

## Abstract

**Background:**

Metachronous gastric tumor (MGT) is one of major concerns after endoscopic submucosal dissection (ESD) for early gastric cancer (EGC). Optimal follow-up strategy has not been yet well-established. The aim of this study was to identify the different clinical features of the patients according to the time interval to development of MGT.

**Methods:**

Among 1,780 consecutive patients with EGC who underwent ESD between 2005 and 2014, 115 patients with MGT were retrospectively reviewed. MGT was defined as secondary gastric cancer or dysplasia detected > 1 year after initial ESD. Clinicopathological factors associated with early development of MGT were evaluated.

**Results:**

The median interval to development of MGT was 37 months. In univariate analysis, the median interval to MGT was shorter if EGC lesion was non-elevated type (39.4 vs 57.0 months, p = 0.011), or synchronous primary lesion was absent (39.8 vs 51.4 months, p = 0.050). In multivariate Cox’s proportional hazards analysis, the hazard ratios for early occurrence of MGT were 1.966 (95% CI: 1.141–3.386, p = 0.015) and 1.911 (95% CI: 1.163–3.141, p = 0.011), respectively. There was no significant difference in overall survival after diagnosis of MGT between the early occurrence group and the late occurrence group.

**Conclusions:**

Non-elevated gross type and absence of synchronous gastric tumor were independent risk factors for early development of MGT. Meticulous endoscopic inspection is especially important for the detection of MGT during the early follow-up period in patients with these initial tumor characteristics.

## Introduction

Gastric cancer is the fifth most common cancer in the world, half of which occurs in eastern Asia. Although the incidence and mortality rates are steadily decreasing worldwide, more than 0.7 million people died of gastric cancer in 2012, making it the third leading cause of death from malignancy [1]. The importance of early detection of gastric cancer cannot be overemphasized, as tumor stage is one of the most powerful prognostic factors [2]. In Korea, more than half of newly detected gastric cancers are in their early stages due to the biennial upper endoscopic examination included in the National Cancer Screening Program [3–5]. Prognosis of early gastric cancer (EGC) is generally excellent after surgical resection, with a 5-year overall survival rate (OS) over 90 percent [6, 7]. Recently, endoscopic resection is widely accepted as a reasonable treatment option in some cases with EGC when the probability of lymph node metastasis is negligible. Data from previous studies show that clinical outcomes such as 5-year OS and recurrence rate after endoscopic submucosal dissection (ESD) are comparable to those after gastrectomy in patients with EGC [8–10]. In addition, ESD is by far a less invasive technique compared to gastrectomy and enables preservation of the whole stomach, which improves quality of life after the procedure [11, 12].

Development of metachronous gastric tumor (MGT) arising from the remnant stomach has always been a major concern after subtotal gastrectomy or endoscopic resection. According to literature, the overall incidence of MGT over a long-term period after endoscopic resection range from 4.3 to 8.5%, while that after partial gastrectomy is about 2.4% [13–16].

Despite the rapid increasing number of patients treated with ESD for EGC, there is no definite consensus on the optimal follow-up strategy. Some studies have shown that the incidence rate of MGT after ESD was not constant over time, and half of events occurred in the first 2–3 years [17, 18]. Considering these findings, investigation of clinical factors associated with interval to development of MGT might affect the follow-up strategy especially in some high-risk patient group. The aim of this study was to evaluate the clinical characteristics and long-term outcomes of patients with EGC according to the time interval to MGT, and associated clinical factors.

## Materials and methods

### Patients

A total of 1,780 consecutive patients who had undergone ESD for EGC at Seoul National University Hospital between January 2005 and June 2014 were retrospectively reviewed. ESD was performed according to the following criteria: well or moderately differentiated adenocarcinoma less than 2 cm in diameter which is confined to mucosa, without evidence of lymph node (LN) or distant metastasis on endoscopic ultrasonography and abdominal computerized tomography. The resection was considered curative if following expanded criteria were fulfilled on the pathologic evaluation of ESD specimens. Expanded criteria were defined as en bloc resection, negative horizontal and vertical resection margin, no evidence of lymphovascular invasion, and including any of the followings: (a) differentiated mucosal cancer without ulceration, regardless of size, (b) differentiated mucosal cancer ≤ 3 cm with ulceration, (c) undifferentiated mucosal cancer ≤ 2 cm, (d) differentiated SM1 (tumor invasion < 500 μm from the muscularis mucosa) cancer ≤ 3 cm. For patients with gastric lesions fulfilling all the other criteria but negative horizontal resection margin, close follow-up strategy with endoscopic biopsy was applied, as the resection margin could be free of tumor because of the electrocauterization effect. In this patient population, if repeated follow-up endoscopy with biopsy revealed no evidence of residual tumor on the previously resected site, the lesion was also regarded as curatively resected.

Patients were excluded if they had previous history of gastric cancer, gastrectomy or endoscopic resection, or underwent subsequent gastrectomy due to non-curative ESD. Tumors containing poorly differentiated, signet ring cell, poorly cohesive, or mucinous carcinoma portion in more than 50% of area were categorized as undifferentiated group. The Institutional Review Board of the Seoul National University Hospital approved this study (IRB number: H-1606-007-765). Patient consent was waived, given the retrospective nature of this study.

### Techniques of endoscopic resection

All ESD procedures were performed using a standard single-channel endoscope (Olympus H260; Olympus Optical), as previously described [19, 20]. In brief, after marking the perimeter 5 mm outside the lesion with several spots using a needle knife (KD- 1L; Olympus) with a forced 20 W coagulation current (VIO 300D; Erbe, Tübingen, Germany), normal saline mixed with indigo carmine and diluted epinephrine (1:100,000) was injected to lift the submucosal layer. Then a small initial incision was made with a needle knife. Subsequent circumferential mucosal incision, followed by dissection of submucosal layer, was made using an insulation-tipped knife (Kachu Technology Co. Ltd., Seoul, Korea).

### Endoscopic and pathologic evaluation

Location and macroscopic type of EGC lesions were assessed according to the Japanese Classification of Gastric Carcinoma (JCGC) [21]. We grouped type 0-I (protruding) and type 0-IIa (superficial elevated) together as elevated type, while grouping type 0-IIb (superficial flat), 0-IIc (superficial depressed), 0-III (excavated), 0-IIa+IIc, and 0-IIb+IIc together as non-elevated type. After ESD, endoscopically resected specimens were immediately stretched and pinned on a flat polystyrene board to prevent folding, and then fixed in 10% formalin. Fixed specimens were then sectioned serially at 2-mm intervals for histologic evaluation. Histologic type, size of tumor, depth of invasion, tumor involvement and lymphovascular invasion were evaluated in accordance with JCGC.

During the ESD procedure, four pieces of non-cancerous gastric mucosal tissue (two from the lesser curvature of antrum and the other two from the lesser curvature of mid body) were obtained by endoscopic biopsy. The tissue samples were examined for histologic evaluation of atrophic change, intestinal metaplasia (IM), and *Helicobacter pylori* status according to the updated Sydney System [22]. The *H*. *pylori* status was evaluated using the rapid urease test (CLO® test; Kimberly-Clark, UT, USA) and histologic examination. If any of these two test results was positive, *H*. *pylori* infection was considered to be present.

### Follow-up schedule

The initial endoscopic follow-up was routinely performed 3 months after ESD. Subsequent follow-up examinations comprising endoscopy, abdominal computed tomography (CT) and chest radiography were scheduled at 6-month intervals for 12 months after ESD, and annually thereafter. Endoscopic biopsy was done on the post-ESD scar or other suspicious mucosal lesions as needed during the follow-up period.

### Definition of synchronous and metachronous gastric tumor

Synchronous and metachronous gastric tumor were defined as second gastric adenocarcinoma or dysplasia detected ≤ 1 year and > 1 year after ESD, respectively. Simultaneous gastric tumors at the time of initial ESD were also interpreted as synchronous lesions.

### Statistical analysis

Pearson’s chi square test or Fisher’s exact test was used to compare categorical variables, and Student’s t-test or Mann-Whitney U test was used to compare continuous variables. The cumulative incidences of MGT by various clinicopathologic factors were evaluated using the Kaplan–Meier method, while the differences between groups were compared with the log-rank test for univariate analysis. Significant univariate factors (*P* < 0.05) and other relevant variables based on previous studies were examined in multivariate Cox proportional hazards regression model to identify independent factors associated with early development of MGT. Results of the analyses were presented as hazard ratios (HRs) with 95% confidence intervals (CIs). *P* value < 0.05 was considered statistically significant. All statistical analyses were performed using SPSS version 23 (SPSS Inc., Chicago, IL, USA).

## Results

### Study population

**Fig 1:**
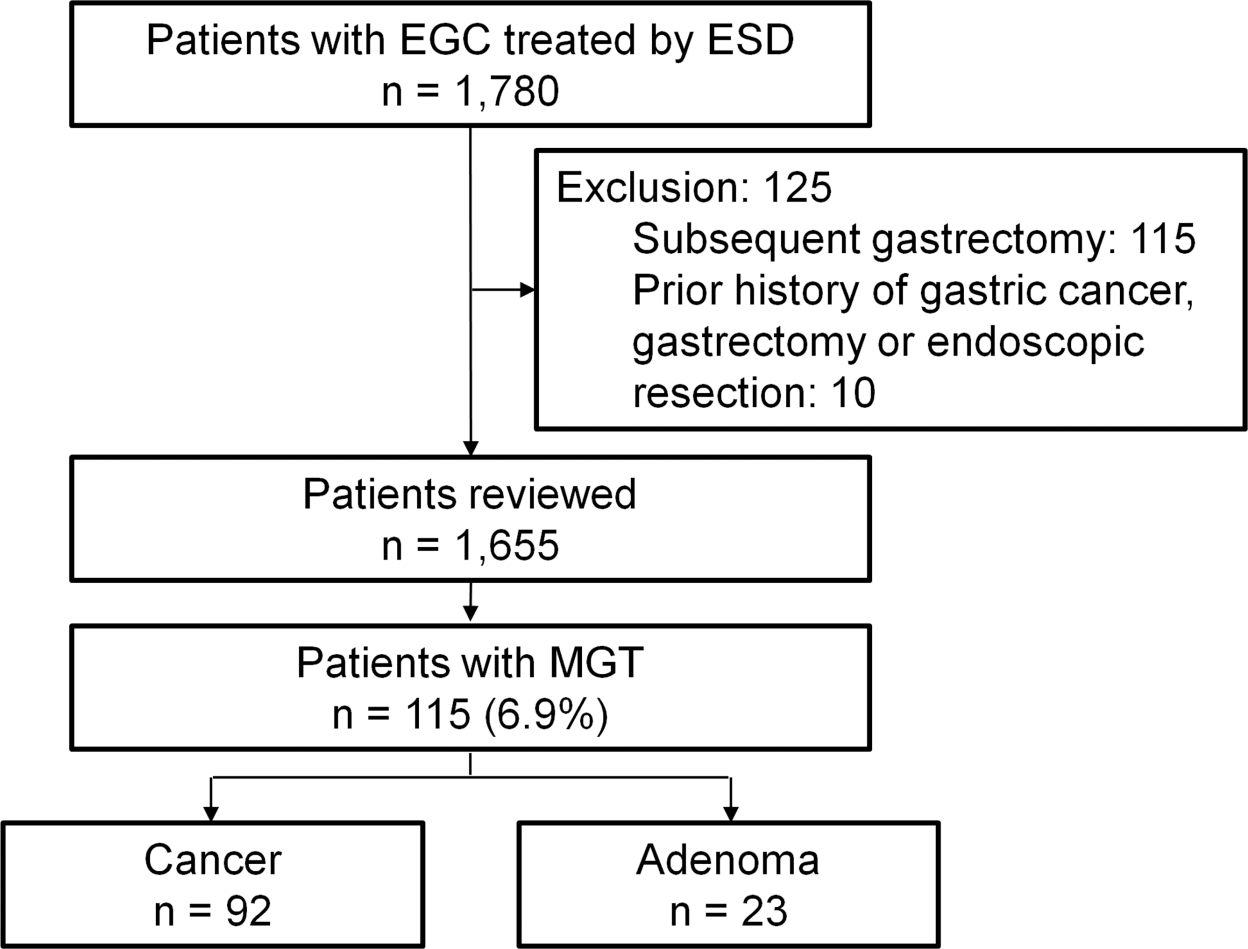
Overview of patient selection process. Inclusion and exclusion criteria for enrolled patients are shown. EGC, early gastric cancer; ESD, endoscopic submucosal dissection; MGT, metachronous gastric tumor.

**Fig 2:**
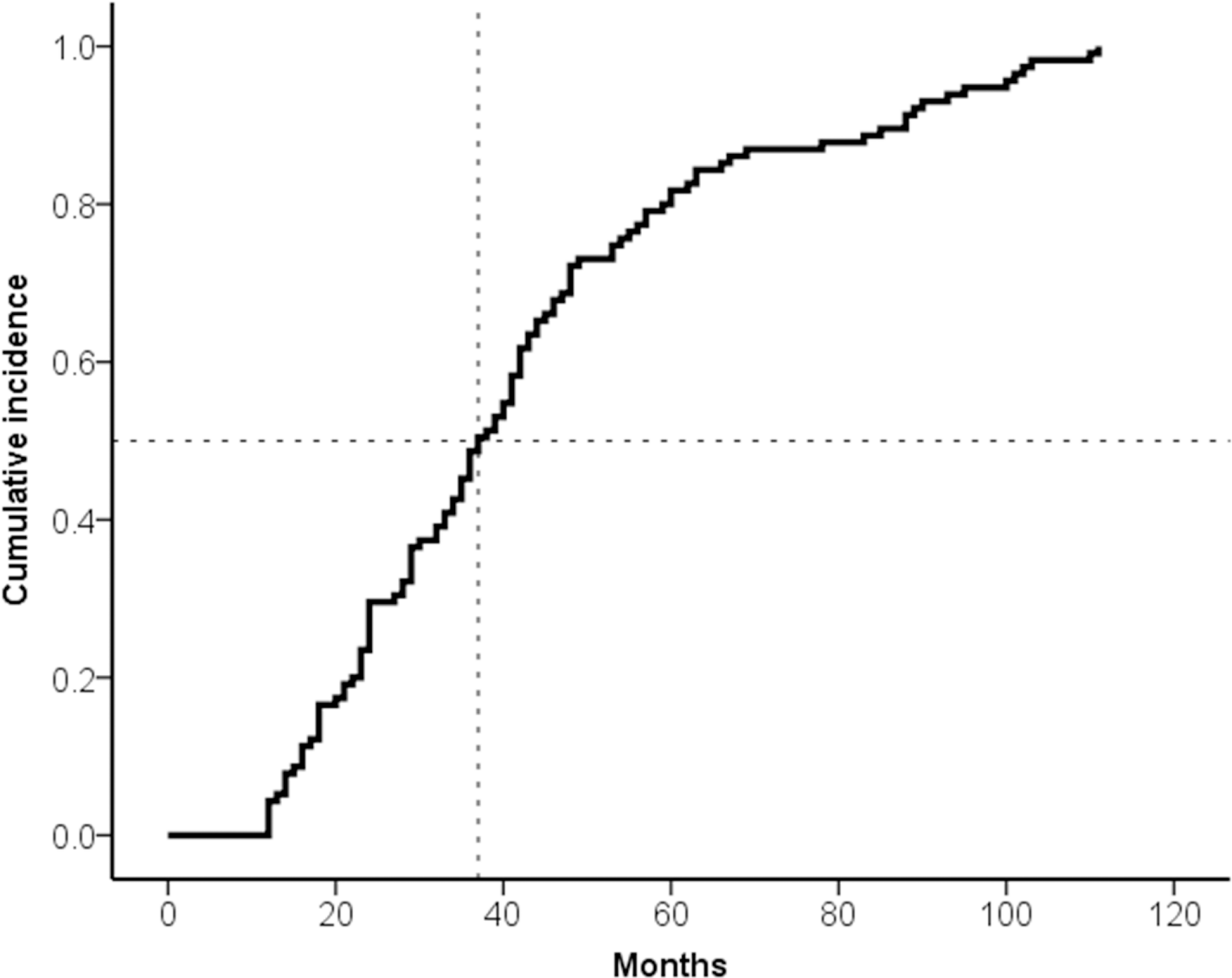
Cumulative incidence of MGT after ESD. This Kaplan-Meier curve shows the trend of development of metachronous gastric tumors after ESD over time.

Among the 1,780 patients who received ESD for EGC, 115 patients who underwent subsequent gastrectomy due to non-curative ESD and 10 patients with prior history of gastric cancer, gastrectomy or endoscopic resection were excluded. Consequently, 1,655 patients were retrospectively reviewed and MGT was confirmed in 115 patients (6.9%, 92 cancers and 23 dysplasias) (Fig 1), while synchronous gastric tumor was found in 31 patients (1.9%, 20 cancers and 11 dysplasias). Among these patients with MGT, trend of tumor development over time was analysed with Kaplan-Meier method (Fig 2). The median time interval to the first MGT was 37 months (interquartile range [IQR], 24–54 months). The mean follow-up duration was 76.9 ± 26.6 months (range: 18–123). Eighty percent of MGTs developed within 60 months from ESD and the longest interval to MGT was 111 month.

### Clinicopathologic features associated with early occurrence of MGT after ESD

Patients with MGT were divided into two groups according to the time interval to occurrence of MGT. As the median time interval to MGT was 37 months, patients diagnosed with MGT within 36 months after the initial ESD were categorized as early occurrence group (n = 52), and the rest were grouped as late occurrence group (n = 63), for the sake of convenience in analysis. The patients with MGT were predominantly male (87.8%), but there was no significant difference in sex, age, body mass index (BMI), and *H*. *pylori* status between the two groups (Table 1). Regarding the initial tumor characteristics, patients of the early occurrence group were more likely to have non-elevated type (p = 0.002), absent or mild IM of antral mucosa (p = 0.002), and less likely to have synchronous gastric tumor (p = 0.042) than those of the late occurrence group. Otherwise, there were no significant differences in curative resection rate, histologic type, location, size, depth of invasion, and presence of coexisting underlying dysplastic lesion between the two groups of patients. Metachronous tumor developed from the previous ESD sites in 13 patients, and from a distant area of the stomach in 102 patients. There were no significant differences in the recurrence site between the early occurrence group and the late occurrence group (6/52 vs 7/63, p = 0.943). Mean follow-up duration was 65.5 ± 26.1 months in the early occurrence group and 86.3 ± 23.3 months in the late occurrence group, respectively (p < 0.001).

**Table 1:** Comparison of clinicopathological characteristics between the early occurrence cases and the late occurrence cases.

Variables	Early occurrence cases (n = 52)	Late occurrence cases (n = 63)	p-value
Mean age ± SD (years)	65.2±8.8	61.8±10.3	0.067
Sex, male	43 (82.7)	58 (92.1)	0.126
Mean BMI ± SD	24.6±2.7	25.0±3.0	0.454
Curative resection	46 (88.5)	55 (87.3)	0.850
Histologic type by Lauren classification			0.729
Intestinal	47 (90.4)	59 (93.7)	
Diffuse or mixed	5 (9.6)	4 (6.3)	
Location			0.761
Upper third	4 (7.7)	4 (6.3)	
Middle third	10 (19.2)	16 (25.4)	
Lower third	38 (73.1)	43 (68.3)	
Gross type			0.002
Elevated type	3 (5.8)	18 (28.6)	
Flat or depressed type	49 (94.2)	45 (71.4)	
Mean size (longest diameter) ± SD (mm)	18.7±1.2	19.2±1.0	0.803
Depth of invasion			0.454
Mucosa	50 (96.2)	58 (92.1)	
Submucosa	2 (3.8)	5 (7.9)	
Coexisting underlying dysplasia	8 (15.4)	14 (22.2)	0.353
AG of mid body mucosa			0.925
Absent to mild / Moderate to marked	17 (51.5) / 16 (48.5)	20 (52.6) / 18 (47.4)	
IM of mid body mucosa			0.624
Absent to mild / Moderate to marked	16 (38.1) / 26 (61.9)	19 (33.3) / 38 (66.7)	
AG of antral mucosa			0.128
Absent to mild / Moderate to marked	17 (65.4) / 9 (34.6)	20 (46.5) / 23 (53.5)	
IM of antral mucosa			0.002
Absent to mild / Moderate to marked	22 (50.0) / 22 (50.0)	12 (20.7) / 46 (79.3)	
Synchronous gastric cancer or dysplasia	8 (15.4)	20 (31.7)	0.042
*Helicobacter pylori* infection	18 (40.9)	24 (40.0)	0.926

Values are number (%) or mean ± SD unless stated otherwise.

SD, standard deviation; BMI, body mass index; AG, atrophic gastritis; IM, intestinal metaplasia.

### Risk factors for early occurrence of MGT after ESD

**Fig 3:**
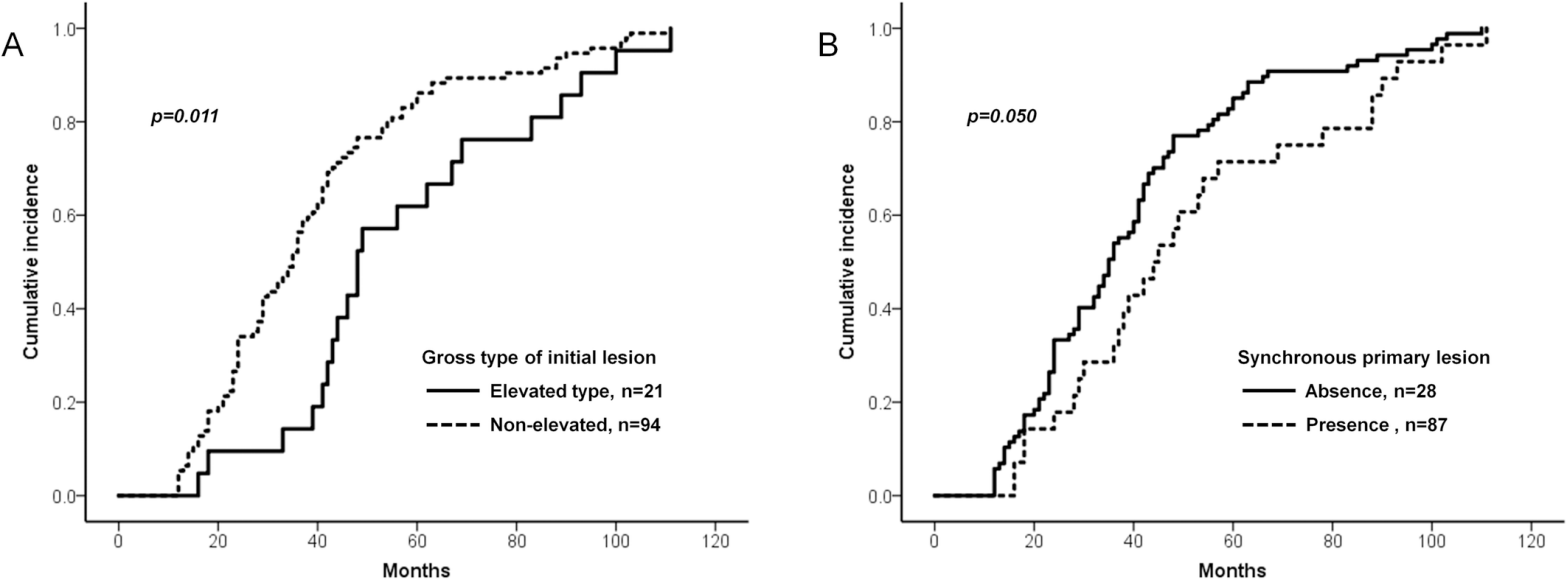
Factors associated with early occurrence of MGT after ESD. (A) Cumulative incidence of MGT by gross type. (B) Cumulative incidence of MGT by presence of synchronous primary lesions.

The univariate analysis using the Kaplan-Meier method and log-rank test showed that non-elevated type at the time of initial ESD was associated with early occurrence of MGT (p = 0.011) (Fig 3A). Absence of synchronous primary lesions also showed almost significant tendency toward early occurrence of MGT (p = 0.050) (Fig 3B). Unlike these factors, age, sex, BMI, curative resection, histologic type by Lauren classification, location, size, depth of invasion, coexisting underlying dysplastic lesion, and severity of atrophic change or IM of mid body and antrum were not significantly associated with early occurrence of MGT (Table 2). In addition to gross type and synchronicity of primary lesion, factors like age, sex, *H*. *pylori* status, and IM of antrum were also analysed in the multivariate Cox proportional hazard model, as they were previously suggested as possible risk factors for MGT.[23–27] In multivariate analysis, non-elevated type (HR 1.966; 95% CI: 1.141–3.386; p = 0.015), absence of synchronous gastric lesion (HR 1.911; 95% CI: 1.163–3.141; p = 0.011) were still independent risk factors for early occurrence of MGT (Table 3).

**Table 2:** Univariate analysis of risk factors for early occurrence of MGT after endoscopic resection of EGC.

Variables	p-value
Age (≥ 65 years)	0.200
Sex (female)	0.950
BMI (≤ 25 kg/m^2^)	0.902
Curative resection (yes)	0.335
Histologic type by Lauren classification (intestinal type)	0.559
Location (upper vs. middle vs. lower)	0.824
Gross type (non-elevated)	0.011
Size (longest diameter) (< 20 mm)	0.304
Depth of invasion (submucosa)	0.679
Coexisting underlying dysplasia (no)	0.538
AG of mid body mucosa (absent or mild)	0.160
IM of mid body mucosa (moderate to marked)	0.562
AG of antral mucosa (absent to mild)	0.334
IM of antral mucosa (absent to mild)	0.236
Synchronous gastric cancer or dysplasia (no)	0.050
*Helicobacter pylori* infection (yes)	0.985

BMI, body mass index; AG, atrophic gastritis; IM, intestinal metaplasia.

**Table 3:** Multivariate analysis of risk factors for early occurrence of MGT after endoscopic resection of EGC.

Variables	HR	95% CI	p-value
Gross type (non-elevated)	1.890	1.104–3.238	0.020
Synchronous gastric cancer or dysplasia (no)	1.606	1.004–2.570	0.048
Sex (male)	1.684	0.849–3.340	0.136
*Helicobacter pylori* infection (yes)	1.973	0.822–1.973	0.279
IM of antral mucosa (absent to mild)	1.255	0.804–1.959	0.318

IM, intestinal metaplasia.

### Effect of H. pylori eradication on development of MGT

*H*. *pylori* status was evaluated in 104/115 (90.4%) patients. Among 42/104 cases (36.5%) with *H*. *pylori* infection, eradication therapy was performed in 15/42 patients (35.7%). Among 15 cases with *H*. *pylori* eradication, successful eradication was confirmed in 11 patients (73.3%). Among those with initial *H*. *pylori* infection, the time interval to MGT was not different between the group with successful *H*. *pylori* eradication (n = 11) and the group with persistent *H*. *pylori* infection (n = 31) (38.2 vs 44.2 months, p = 0.245). Excluding 2 patients who lacked clinical information of atrophic change or IM of stomach, 38 out of 40 patients (95.0%) had either atrophic gastritis or IM in antrum or mid body. Kaplan-Meier analysis of the cumulative incidence of MGT in time-dependent manner and log-rank test revealed no statistical difference between the two groups (p = 0.502).

### Characteristics of MGT

Among 115 patients with MGT, metachronous lesion was cancer in 92 patients (80.0%), and dysplasia in 23 patients (20.0%). Gastric cancer was advanced lesion in 4/92 patients (4.8%) and EGC in 80/92 patients (87.0%). The other 8 patients could not be assessed, as they had only extragastric lesions or did not undergo resection of the lesion due to severe comorbidities, or refused further evaluation. Characteristics of MGT lesions were compared between the early occurrence group and the late occurrence group. Among 52 patients with early MGT and 63 patients with late MGT, metachronous lesion was cancer in 40 patients and 52 patients, respectively, and the proportions were not different between the two groups (p = 0.454). Regarding the patients with only intragastric metachronous cancer, differentiated type histology was present in 32/39 patients with early MGC, and in 39/50 patients with late MGC (p = 0.637). Proportion of elevated type (4/35 vs 6/44, p = 1.000) was also not different between the two groups of patients.

### Long-term follow-up results and prognosis in patients with MGT

Among 115 patients with MGT, metachronous lesions were treated with repeated ESD in 87 patients (75.7%), surgical resection in 22 patients (19.1%), and chemotherapy in 1 patient (0.87%). Regarding the remaining 5 patients, one patient was carefully observed without definitive treatment for MGT because of underlying metastatic hepatocellular carcinoma, and the other 4 patients did not show up after the diagnosis of MGT.

**Fig 4:**
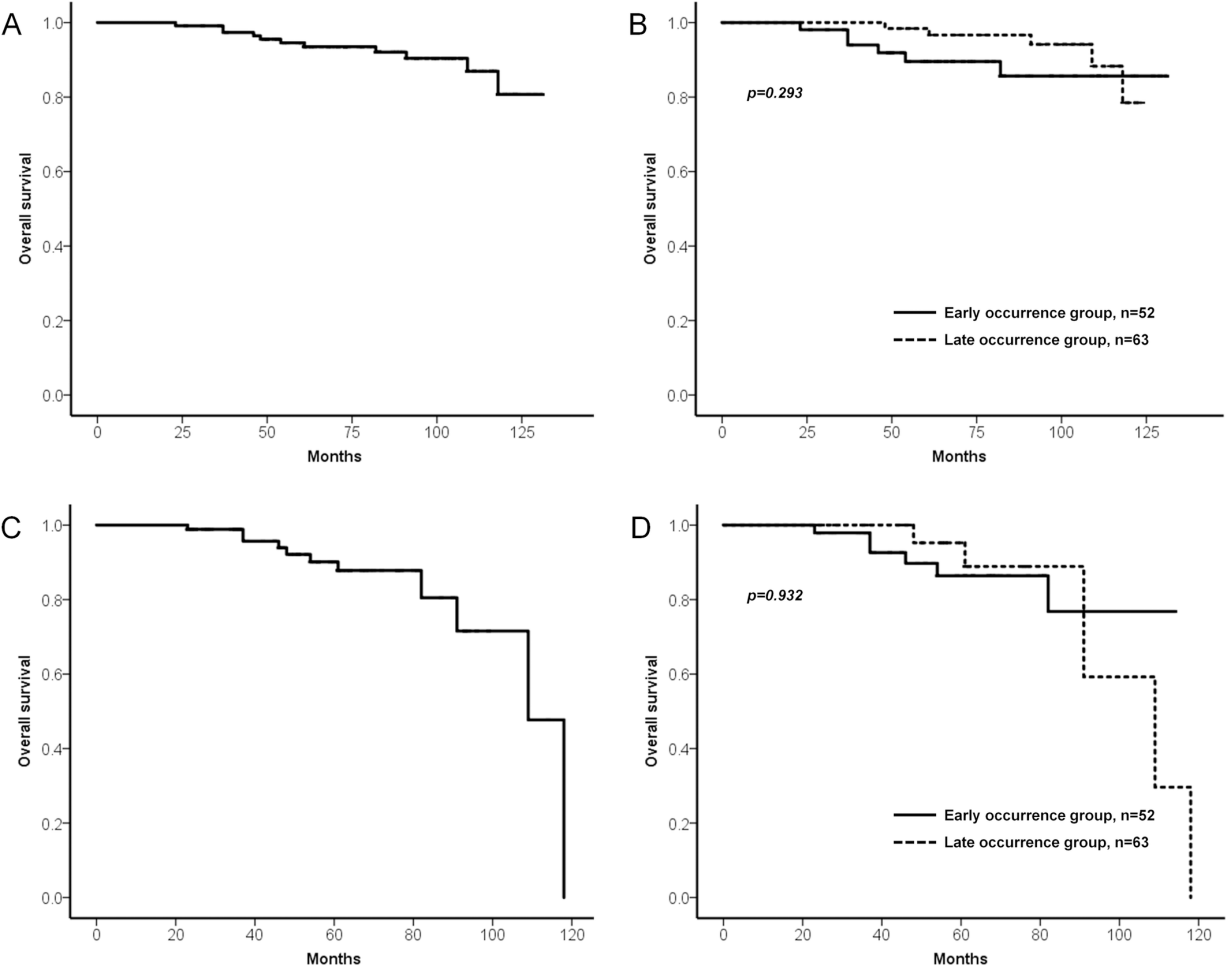
Survival analysis of the patients with MGT after ESD. (A) Kaplan-Meier survival curve for overall survival in the whole patient group. (B) Kaplan-Meier survival curves for overall survival in early occurrence group and late occurrence group. (C) Kaplan-Meier survival curve for overall survival after diagnosis of MGT in the whole patient group. (D) Kaplan-Meier survival curves for overall survival after diagnosis of MGT in early occurrence group and late occurrence group.

Among 115 patients, 11 patients (9.6%) died during a median follow-up period of 7.3 years (IQR, 5.1–8.9 years). In Kaplan-Meier curve for overall survival, the 5-year, 7-year, and 10-year OSs for the whole group of patients were 93.4%, 90.3%, and 81.3%, respectively (Fig 4A). Subsequent log-rank test showed that there was no significant difference in overall survival between the early occurrence group and the late occurrence group (Fig 4B). In addition, the 5-year OS after diagnosis of MGT was 87.2% (Fig 4C), and there was no significant difference in overall survival after diagnosis of MGT between the two groups (Fig 4D). Cause of death was gastric cancer in 3 patients (2.6%), and the 10-year disease-specific survival rate was 97.3%. The time intervals to MGT were 17, 35, and 40 months in these 3 patients, respectively.

## Discussion

As ESD is frequently performed for treatment of differentiated mucosal EGC especially in East Asia nowadays, the patients with EGC can maintain better quality of life after ESD than after gastrectomy via reservation of the whole stomach. At the same time, MGT arising from the remnant stomach is undoubtedly one of the most concerning issues after ESD because this may be directly related to poor prognosis. While accumulated experiences with endoscopic resection over nearly two decades has led to the consensus of indication for the procedure, when to discontinue endoscopic follow-up is still a debatable issue. Focusing on this specific subject, we investigated the clinicopathologic factors associated with the time interval to between the development of MGT and the initial ESD.

In this study, the 5-year cumulative incidence of MGT was 5.6% and overall cumulative incidence was 6.9% over the mean follow-up duration of 76.9 month. These incidence rates are lower compared to those observed in previous studies [13–15]. This difference may be attributable to *H*. *pylori* eradication therapy applied after ESD to some of the patients with *H*. *pylori* infection, considering the protective effect of eradication therapy against development of MGT [23]. The incidence rate of MGT was not constant over time after ESD. Considering half of MGT occurred in the 2nd or 3rd year after the procedure, meticulous endoscopic inspection is required after ESD especially in the early follow-up period. In addition, as one fifth of MGT occurred later than 5 years after ESD, long-term follow-up might be beneficial to some patients with adequate life expectancy without severe comorbidities.

There have been some studies suggesting the association between endoscopic gross appearances and clinical behaviour of EGC [28, 29]. A total of 546 (35%) patients had elevated lesion among 1,780 cases undergoing ESD for EGC in our study, compared to 15.9–19.8% in previous studies with surgically treated EGC patients [28, 30, 31]. This might be attributable to the difference of target patient groups, as overt ulceration or undifferentiated histology was contraindication for ESD. Additionally, considering that elevated gross type is directly associated with differentiated type histology, this association may explain why elevated type was taking relatively large proportion than expected [32]. Meanwhile, 21/115 (18.3%) patients with MGT had elevated initial lesion, and only 3 out of 21 patients with elevated type EGC (14.3%) had occurrence of MGT in 3 years. As epigenetic change with microsatellite instability seems to have a role in growth pattern of EGC,[33] methylation induced suppression of DNA mismatch repair genes might be a factor affecting the time interval to development of MGT.

Absence of synchronous gastric tumor was another independent risk factor for early occurrence of MGT. Detection of synchronous gastric tumor is largely dependent on the skill and experience of the endoscopist as well as the location or size of the lesion, and also reflects how meticulous the examination was done. To be detectable on the endoscopic examination, neoplastic lesion should grow large enough over time. This means there is still some chance of missed small EGCs or precancerous lesions after an endoscopic examination. Although we have categorized gastric tumor detected simultaneously with the index EGC or in the first year after ESD as synchronous tumor due to the possibility of overlooked lesion, some of the early MGTs might still be the consequences of initially missed neoplastic lesions. If coexisting precancerous lesions or small EGC lesions were detected initially and resected together with the index EGC, it can be inferred that new gastric neoplastic lesion would rarely be found in the next few years. Therefore, presence of synchronous gastric tumor would be a factor partially reflecting how precisely the endoscopic evaluation was performed and whether precancerous lesions were removed initially.

As the study population encompassed post-ESD patients over 10 years, *H*. *pylori* eradication therapy was individually applied to each of the patient considering several factors like age, comorbidities or patient’s preference. Among 42 *H*. *pylori*-infected patients, successful eradication was achieved in 11 patients and the time interval to the first MGT was not different between the two groups with and without persistent *H*. *pylori* infection. We also observed that almost every patient with initial *H*. *pylori* infection had atrophic change or IM in some part of the stomach. Although the number of patients who achieved successful eradication was rather small, *H*. *pylori* infection or eradication did not affect the time interval to occurrence of MGT after ESD. This finding, which is similar to the result from the previous study by Lim et al.,[13] implies that *H*. *pylori* eradication therapy may not delay or prevent the progression of malignant change, especially when the neoplastic change has reached a certain degree. In addition to *H*. *pylori* status, sex and IM were not associated with early occurrence of MGT. We also compared various characteristics of MGT such as histology, differentiation, and gross type between the early occurrence group and the late occurrence group, which showed no significant differences between the two groups. Although statistically insignificant, the mean age was lower in the late occurrence group than in the early occurrence group. This is closely associated with the fact that elderly patients are more likely to die from various causes other than gastric cancer. In young patients, late MGTs are more common than in old patients because of relatively long survival time and long follow-up duration.

Both the 5-year OS after initial ESD and the 5-year OS after diagnosis of MGT were over 85% in this study, which are comparable to the same figure of patients with EGC, showing that patients who once underwent ESD for EGC have excellent prognosis even after the diagnosis of MGT if adequately treated. In addition, there was no statistical difference in overall survival between the early occurrence group and the late occurrence group, implying that long-term prognoses of the two groups of patients were not significantly different.

As high grade dysplasia is widely considered as premalignant lesion, it is reasonable to count this lesion as MGT. Although low grade dysplasia has relatively low risk of progression to malignant lesion, pathologic discrepancies commonly exist between the specimens obtained with single endoscopic biopsy and resected specimens as a whole. Due to this histologic heterogeneity of tumor, endoscopic resection of low grade dysplasia is generally recommended. Considering these issues in clinical practice, dysplasia of any degree was included in MGT in our study. To the best of our knowledge, this is the first study to investigate the association between the time interval to MGT and the prognosis of patients with EGC treated by ESD, as well as risk factors for early occurrence of MGT. An additional strength of this study was the availability of long term follow-up data. The mean follow-up duration was over 6 years, and every patient included in the analysis had follow-up duration longer than 18 months.

This study has some limitations. As a restrospective, single-center study, the result should be cautiously interpreted and adapted in general population. Possibility of some degree of bias could not be ruled out due to its retrospective nature. For this reason, we tried to minimalize possible biases through multivariate analysis. Although atrophic gastritis and IM was not evaluated in every patient, the histologic change of gastric mucosa was examined and graded by pathological review, which is more reliable method than mere macroscopic inspection. Regarding *H*. *pylori* status, we reviewed two different tests including histologic examination to avoid false negative results, but individual history of previous *H*. *pylori* eradication before the ESD procedure was not available.

In conclusion, non-elevated type and absence of synchronous gastric tumor were independent risk factors for early development of MGT. These factors can be interpreted as being related with missed gastric tumors at initial endoscopic examination. Meticulous endoscopic inspection is required after ESD for EGC, especially in early follow-up periods. Long-term follow-up is also recommended regardless of *H*. *pylori* status in patients with adequate life expectancy without severe comorbidities.
